# Supplementary material for: Bacterial supplementation shapes honey bee gut microbiota and host metabolism under controlled and field conditions
Source: mSystems. 2026 May 21;11(6):e00283-26. doi: 10.1128/msystems.00283-26 (PMC13289173; doi:10.1128/msystems.00283-26)
Supplement: Supplemental information — Supplemental methods, Figures S1-S3, and abbreviations and full names. [file msystems.00283-26-s0001.docx]

**Supplementary Information of**

**Bacterial supplementation shapes honey bee gut microbiota and host metabolism under controlled and field conditions**

This document contains:

Supplementary methods

Figures S1-S3

Abbreviations and full names

References

**Supplementary methods**

1. **Bacterial mixture preparation**

The strains were isolated from the digestive tract of honey bees originating from the three locations in the Czech Republic (D1 strains: Dol-Máslovice, 50.2039797N, 14.3667811E; P1/MR12: Postřižín, 50.2267222N, 14.3778611E; VT5: Větrušice, 50.1895211N, 14.3821864E). The selective Rogosa agar for lactobacilli, M.R.S. agar (both Oxoid, UK) for lactic acid bacteria, and modified TPY agar for bifidobacteria were used for the isolation process by the anaerobic microbiological techniques, as described previously (1).

Fresh 18–24-hour cultures were inoculated into modified Brain Heart Infusion (mBHI) medium under anaerobic conditions (2% v/v; anaerobiosis maintained using a gas mixture of 75% N₂, 20% CO₂, and 5% H₂) and incubated at 37 °C for 18–24 hours until reaching the late logarithmic growth phase (2). The mBHI medium was composed (g/L) by BHI (Carl Roth, DE): 37, glucose: 8, yeast extract (Oxoid, UK): 3, soybean peptone: 3, meat extract: 2 (both Carl Roth, DE), KH_2_PO_4_: 2, MgCl_2_: 0.5, and 0.5 mL of Tween 80 (Thermo Fisher Scientific, USA). The pH was adjusted to 7.1-7.3 with 10M NaOH before sterilization (107 °C, 50 min). The cultures were then mixed and centrifuged (6000 rpm, 6 min). The bacterial pellets were rinsed with anaerobic sucrose solution (5%, w/v) and centrifuged again under the same conditions. Then, the pellets were again resuspended in the sucrose solution and proportionally distributed in conical plastic tubes. Bacterial suspensions were immediately frozen at -85 °C for 4 h and freeze-dried (L10-55 PRO, Gregor instruments, CZ) for 24 h. The total cell concentration after lyophilization in three randomly selected samples was calculated by anaerobic plate technique using the mBHI agar. Serially diluted samples were incubated in the 3.5L anaerobic jar (Oxoid, UK) at 37 °C for 72 h.

The probiotic mixture consisted of five bacterial strains: D1/RO2, D1/MR10/B2, D1/MR10/B4, P1/MR12, and VT5. Based on 16S rRNA gene sequence analysis, the strains were identified as *Lactobacillus helsingborgensis* (GenBank accession number PP754626; 99.86% homology to type strain Bma5^T), *Bifidobacterium polysaccharolyticum* (PP754621; 99.64% to W8117^T), *B. choladohabitans* (PP754622; 100% to B14384H11^T), *L. helsingborgensis* (PP754641; 99.93% to Bma5^T), and *L. apis* (PP754656; 99.93% to R4B^T), respectively (3).

The viable cell concentrations in the individual culture stocks were 1.244 × 10¹⁰ (D1/RO2), 2.79 × 10¹⁰ (D1/MR10 co-culture of B2 and B4), 3.13 × 10⁹ (P1/MR12), and 2.418 × 10⁹ cells/mL (VT5). The final bacteria formulation used per hive equaled a tube content and contained approximately 27.1% *L. helsingborgensis* (D1/RO2), 30.4% *B. polysaccharolyticum* (D1/MR10/B2), 30.4% *B. choladohabitans* (D1/MR10/B4), 6.8% *L. helsingborgensis* (P1/MR12), and 5.3% *L. apis* (VT5).

The final bacterial counts of the freeze-dried mixture in individual tube were 4.62 × 10^10^ CFU (Colony Forming Units). This amount was used per single hive treatment or was correspondingly diluted to 1.00 × 10^9^ as a treatment for the cage experiment.

1. **Bacteriome profiling**
   1. **Amplicon sequencing**

The bacteriome profiling was performed using next-generation amplicon sequencing of a PCR product spanning the V3 and V4 regions of the 16S rRNA gene. The tagged specific primer sequences, 341-F (4) and 806-R (5) were synthesized with additional bases that increase the sequencing signal heterogeneity (O. Cinek *et al*., accepted for publication; the sequences, a detailed protocol and bioinformatic scripts available at https://github.com/ondrejcinek/primer_spacers). Each sample was amplified in a duplicate of reactions using high-fidelity DNA-dependent DNA polymerase. The two reactions differed by the orientation of the sequencing adaptors to further increase signal heterogeneity. After reaction clean-up, samples were indexed using a limited count of PCR cycles with Illumina Nextera combinatorial index primers (sets A and D). Indexed PCR products were purified again using AMPure magnetic beads, equalized, and sequenced on the Miseq platform (Illumina, USA) using the V2 kit at 2 × 250 bp, with an addition of 10% PhiX library (Illumina). The run yielded 14.5 million clusters, the Q30 threshold was exceeded by 94.1% forward, and 86.5% reverse reads.

- 1. **Processing of 16S rRNA gene amplicon-sequencing data**

The raw data were downloaded as demultiplexed fastq files with trimmed adapters; the heterogeneity spacers were trimmed, and orientation of amplicons unified, using a custom Python script available in the above repository along with a brief user guide (github.com/ondrejcinek/primer_spacers). Reads of the same fragment were merged using USEARCH (6). After quality inspection, data were processed in the DADA2 pipeline (7). Reads were filtered with maximum allowed number of expected error equal to 2, demultiplexed, chimerae removed, amplicon sequence variants (ASV) counted and taxonomically classified using the BEExact database (8), version 2023.01.30, V3V4 fragment formatted for DADA2, downloaded from github.com/bdaisley/BEExact on 1/3/2023. Phylogenetic tree of ASVs was constructed using FastTree2 (9) with the generalized time-reversible model of evolution and gamma20 likelihood. Negative controls were checked for the absence of a significant signal, and the mock community positions were assessed for agreement with their declared content. To verify agreement between replicates, an ordination plot was created by principal coordinate analysis of the Bray-Curtis dissimilarity among individual reactions; after its inspection, duplicates were merged by summing counts. After inspection, replicate samples were merged by summing counts. Phyloseq objects were then created by agglomerating taxonomic data at several levels: phylum, class, order, family, genus, and the ASV level. Finally, taxonomic units that are not present in at least 80% of the samples were removed. Data were rarefied to 14,499 counts per sample across all taxonomic levels.

1. **Proteome analysis**
   1. **Sample processing**

Bee guts were homogenized in 300 µL of lysis buffer (6 M urea, 2 M thiourea, and Complete™ Mini Protease Inhibitor Cocktail, Roche, CH) using a handheld motorized pestle. After centrifugation (9,000 × g, 5 min), protein concentrations were determined using the Qubit™ Protein Assay Kit (Invitrogen, USA) on a Qubit fluorometer (Thermo Fisher Scientific, USA).

75 µL of each protein extract were purified with a 2-D Clean-Up Kit (Cytiva, USA) following the manufacturer’s instructions. Pellets were resuspended in 90 µL of buffer (6 M urea, 2 M thiourea, 0.1 M Tris-HCl, pH 8.0), and 20 µL was used for digestion. To each sample, 105 µL of 50 mM ammonium bicarbonate was added, and proteins were reduced with 0.5 M dithiothreitol at 56°C for 20 min and alkylated with 0.5 M iodoacetamide for 15 min in the dark at room temperature. ProteaseMax™ (1% w/v, Promega, USA) and sequencing-grade trypsin (Promega, USA) were added (1 µL each), and samples were incubated overnight at 37°C. Digestions were terminated by the addition of 1 µL trifluoroacetic acid followed by centrifugation (13,000 × g, 10 min).

Peptides were purified using C18 spin columns (Thermo Fisher Scientific, USA) following the manufacturer’s instructions, dried under vacuum, and resuspended in 2% acetonitrile/0.05% TFA to a final concentration of 0.5 µg/µL. After vortexing, sonication (3 min), and centrifugation (13,400 rpm, 5 min), supernatants were used for LC-MS/MS.

- 1. **LFQ mass spectrometry analysis and data processing**

For each sample, 1 µg of peptide was injected into a Q Exactive mass spectrometer (Thermo Fisher Scientific, USA) coupled to a Dionex Ultimate 3000 nano LC system. Peptides were separated on a Biobasic C18 Picofrit™ column (200 mm × 75 µm ID) using a 120-minute acetonitrile gradient at 250 nL/min. The mass spectrometer operated in data-dependent acquisition mode, selecting the 15 most intense ions for MS/MS after each full MS scan (m/z 300–2000).

MaxQuant v2.4.2.0 was used for protein identification and LFQ normalization of all MS/MS data. The Andromeda search algorithm in MaxQuant was used to correlate all MS/MS data against protein reference sequences obtained from the National Centre for Biotechnology to correlate the data against the protein reference sequences for *A. mellifera* genome obtained from the National Centre for Biotechnology Information (NCBI) repository (23,520 entries, downloaded June 2023). The mass spectrometry proteomics data have been deposited to the ProteomeXchange consortium via the PRIDE partner repository (10) with the data set identifier PXD066488.

LFQ data were processed using Perseus (v2.0.10.0). Initial filtering removed contaminants, reverse hits, and site-only identifications. Log2 transformation was applied to LFQ intensities. Proteins not detected in all replicates of at least one group were excluded. Missing values were imputed using a downshift of 2.1 times and a width of 0.1 times the standard deviation of all measured values, simulating low-abundance signals. A total of 977 proteins were retained for further statistical analysis (Table S3).


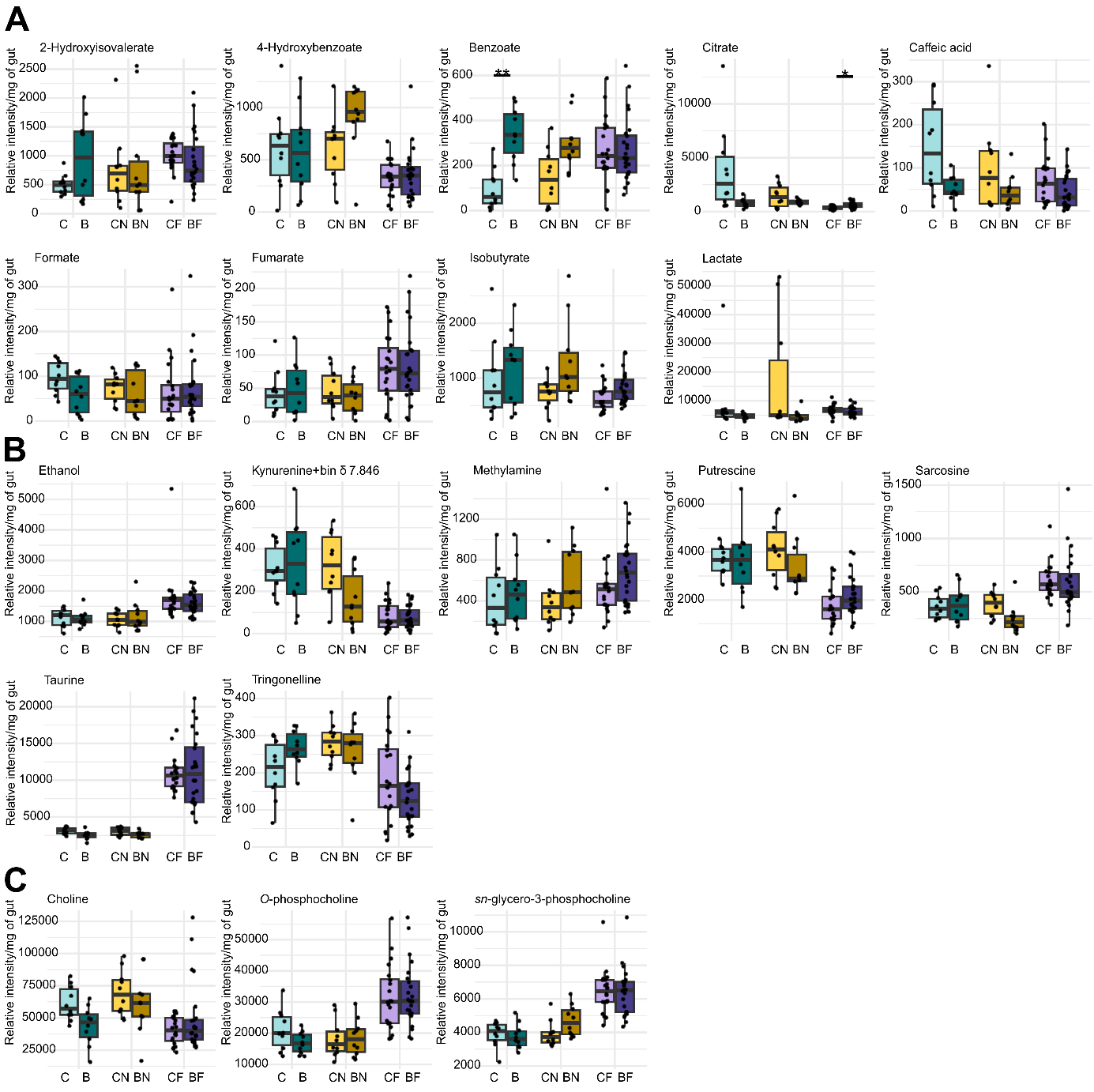


**Figure S1.** **Metabolic profiling of honey bee gut samples across experimental groups in cage and field experiments. Detected metabolites are categorized and presented as follows: (A) organic acids, (B) amines and others, and (C) lipid metabolism-related compounds.** Box plot visualization and statistical analysis between C vs. B, CN vs. BN, and CF vs. BF. Statistically significant adjusted *P*-values are indicated with asterisks mark (* *q* < 0.05, ** *q* < 0.01, no marks = ns). Statistical comparisons were performed using the Wilcoxon test, followed by the Benjamini-Hochberg procedure (C, B, CN, BN; *n* = 10 per group; CF, *n* = 20; BF, *n* = 24). Group abbreviations: C, control (cage); B, bacterial supplementation (cage); CN, control with nestmates (cage); BN, bacterial supplementation with nestmates (cage); CF, control (field); BF, bacterial supplementation (field).


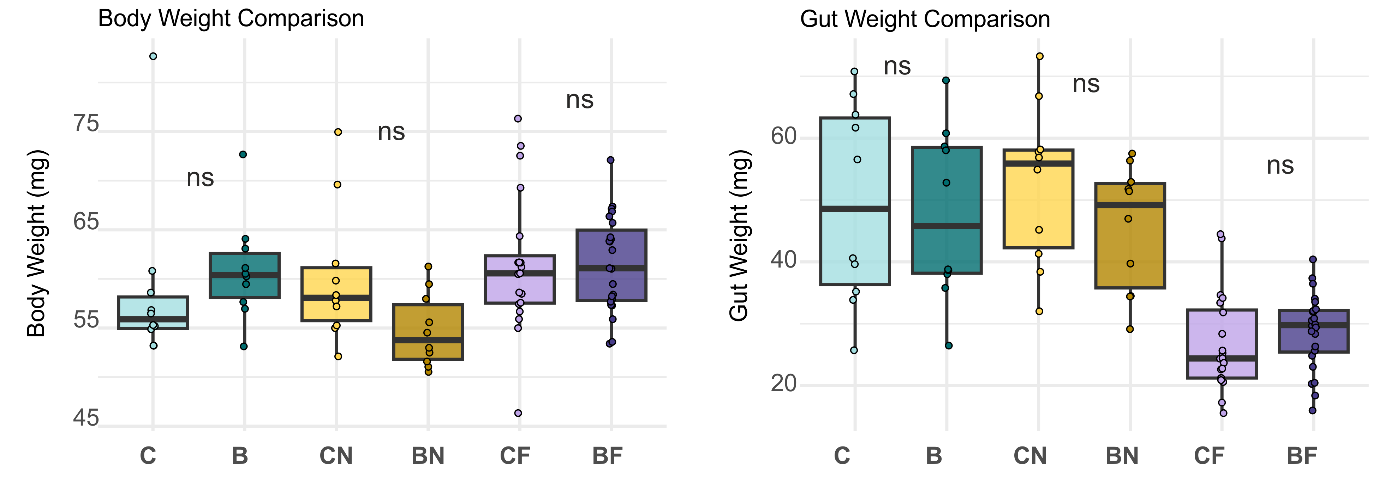


**Figure S2. Comparison of honey bee body (headless) and gut weights (digestive tract apart from the crop) across experimental groups.** Box plot analysis of metabolites between C vs. B, CN vs. BN, and CF vs. BF. Statistical comparisons were performed using the Wilcoxon test (C, B, CN, BN; *n* = 10 per group; CF, *n* = 20; BF, *n* = 24). Group abbreviations: C, control (cage); B, bacterial supplementation (cage); CN, control with nestmates (cage); BN, bacterial supplementation with nestmates (cage); CF, control (field); BF, bacterial supplementation (field).


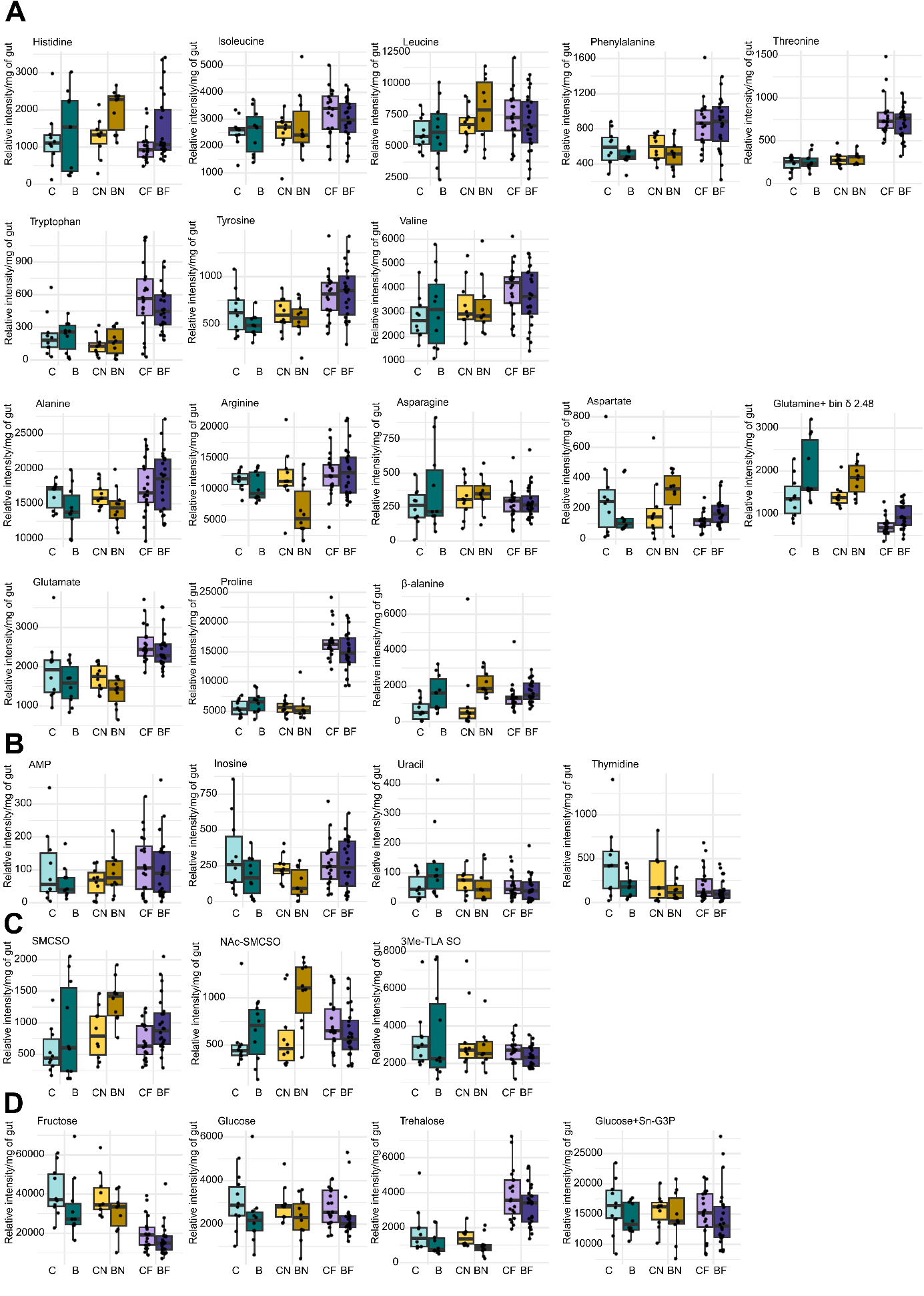


**Figure S3.** **Metabolic profiling of honey bee gut samples across experimental groups in cage and field experiments. Detected metabolites are categorized and presented as follows: (A) amino acids, (B) purine/pyrimidine metabolism (C) sulphur-containing compounds (D) carbohydrates.** Box plot visualization and statistical analysis between C vs. B, CN vs. BN, and CF vs. BF. Statistically significant adjusted *P*-values are indicated with asterisks mark (* *q* < 0.05, ** *q* < 0.01, no marks = ns). Statistical comparisons were performed using the Wilcoxon test, followed by the Benjamini-Hochberg procedure (C, B, CN, BN; *n* = 10 per group; CF, *n* = 20; BF, *n* = 24). Group abbreviations: C, control (cage); B, bacterial supplementation (cage); CN, control with nestmates (cage); BN, bacterial supplementation with nestmates (cage); CF, control (field); BF, bacterial supplementation (field).

**Abbreviations and full names**

3Me-TLA SO: 3-methylthiolactic acid sulfoxide

4-HBA: 4-Hydroxy benzoic acid

Ala: Alanine

Arg: Arginine

Asn: Asparagine

Asp: Aspartic acid

DAT: Desaminotyrosine (3-(4-Hydroxyphenyl)propionic acid)

DHCA: Dihydrocaffeic acid (3,4-Dihydroxyhydrocinnamic acid)

GABA: γ-aminobutyric acid

GlcNAc: *N*-Acetylglucosamine

Gln: Glutamine

Glu: Glutamate

His: Histidine

Ile: Isoleucine

Lys: Lysine

NAc-SMCSO: *N*-acetyl-*S*-methyl-L-cysteine sulfoxide

Orn: Ornithine

Phe: Phenylalnine

Pro: Proline

SMCSO: *S*-methyl-L-cysteine sulfoxide

sn-G3P: *sn*-glycerol-3-phosphate

Thr: Threonine

Trp: Tryptophan

Tyr: Tyrosine

Val: Valine

**References**

1. Killer J, Kopečný J, Mrázek J, Rada V, Dubná S, Marounek M. 2010. Bifidobacteria in the digestive tract of bumblebees. Anaerobe 16:165–170.

2. Killer J, Bunešová VN, Modráčková N, Vlková E, Pechar R, Šplíchal I. 2023. Lactulose in combination with soybean lecithin has a cryoprotective effect on probiotic taxa of bifidobacteria and *Lactobacillaceae*. Lett Appl Microbiol 76.

3. Dengiz B, Killer J, Havlik J, Dobes P, Hyrsl P. 2025. Selection of probiotics for honey bees : the *in vitro* inhibition of *Paenibacillus* *larvae* , *Melissococcus* *plutonius*, and *Serratia* *marcescens* strain sicaria by host-specific Lactobacilli and Bifidobacteria. Microorganisms 13:1–17.

4. Muyzer G, De Waal EC, Uitterlinden AG. 1993. Profiling of complex microbial populations by denaturing gradient gel electrophoresis analysis of polymerase chain reaction-amplified genes coding for 16S rRNA. Appl Environ Microbiol 59:695–700.

5. Caporaso JG, Lauber CL, Walters WA, Berg-Lyons D, Lozupone CA, Turnbaugh PJ, Fierer N, Knight R. 2011. Global patterns of 16S rRNA diversity at a depth of millions of sequences per sample. Proc Natl Acad Sci U S A 108:4516–4522.

6. Edgar RC. 2010. Search and clustering orders of magnitude faster than BLAST. Bioinformatics 26:2460–2461.

7. Callahan BJ, McMurdie PJ, Rosen MJ, Han AW, Johnson AJA, Holmes SP. 2016. DADA2: High resolution sample inference from Illumina amplicon data. Nat Methods 13:518–583.

8. Daisley BA, Reid G. 2021. BEExact: a metataxonomic database tool for high-resolution inference of bee-associated microbial communities. mSystems 6.

9. Price MN, Dehal PS, Arkin AP. 2009. FastTree: Computing large minimum evolution trees with profiles instead of a distance matrix. Mol Biol Evol 26:1641–1650.

10. Perez-Riverol Y, Bandla C, Kundu DJ, Kamatchinathan S, Bai J, Hewapathirana S, John NS, Prakash A, Walzer M, Wang S, Vizcaíno JA. 2025. The PRIDE database at 20 years: 2025 update. Nucleic Acids Res 53:D543–D553.
